# Have changes in colorectal surgery training impacted on mortality in cancer patients? A retrospective cohort study of 51,562 procedures

**DOI:** 10.1308/rcsann.2024.0059

**Published:** 2024-09-03

**Authors:** AG Taib, Z Patel, A Kler, CA Maxwell-Armstrong

**Affiliations:** ^1^ The Royal Blackburn Teaching Hospital, UK; ^2^ University Hospital Morecambe Bay NHS Foundation Trust, UK; ^3^ Furness General Hospital, UK; ^4^ Nottingham University Hospitals NHS Trust, UK

**Keywords:** colorectal surgery, continuing medical education, patient outcome assessment, colorectal cancer, training programs

## Abstract

**Introduction:**

The aim of this study was to explore whether there were any differences in consultant colorectal surgeon training and adjusted 90-day postoperative colorectal cancer mortality rates (AMR).

**Methods:**

We undertook a retrospective analysis of outcomes data published on the Association of Coloproctology of Great Britain and Ireland (ACPGBI) website. A total of 51,562 procedures for patients in England diagnosed with large bowel cancer between 2010 and 2015, registered under 551 consultants were included. Consultants were split into two cohorts. The first group were the pre-Calman Trained Consultants (pre-CTr), who completed their training before 1998. The second group—the post-Calman Trained Consultants (post-CTr)—included those who received their Certificate of Completion of Training (CCT) under the Calman Training Principles (CTC, 1998–2007) and the Modernising Medical Careers Curriculum (MMC, 2008 and onwards). The outcome measure was an AMR.

**Results:**

The pre-CTr cohort (*n*=84) consisted of 3.6% female colorectal consultants (*n*=3/84), whereas the post-CTr cohort (*n*=467) consisted of 14.3% female colorectal consultants (*n*=67/467) (*p*=0.006). In this cross-sectional analysis over 5 years, the average pre-CTr undertook a greater number of colorectal resections than their post-CTr peers: median procedures (interquartile range, IQR): 104 (59) vs 89 (57) respectively, *p*=0.008. The median AMR was significantly greater among pre-CTrs compared with post-CTrs, median AMR (IQR): 2.7% (2.0) vs 2.1% (2.9), *p*=0.022.

**Conclusions:**

These data indicate that the implementation of the MMC and Calman training principles for colorectal training is associated with a statistically lower AMR compared with other historical training periods. This merits further exploration.

## Introduction

Surgical training has changed significantly in the UK over the past 20 years. The requirement for surgical trainees to fundamentally become independent consultant practitioners remains the same. However, for today's surgeons, complexity has increased and expectations are higher.

Originally, surgery was an apprenticeship. Surgeons rotated through smaller and larger centres throughout the country, acquiring practical and theoretical knowledge from seniors. Progress was dictated by the opinions of senior surgeons and consultants formally and informally. The Fellowship of the Royal College of Surgeons exam ensured all trainees had a standardised knowledge base before consultancy, though there was no ‘exit’ examination. However, there was a lack of educational framework and formalised assessment of technical and non-technical skills. This translated to a vague and often lengthy surgical training.

In 1997, Kenneth Calman, the Chief Medical Officer for England, implemented the ‘Calman’ principles. These proposals changed training in all specialties. The key aim was to ‘produce a shorter, more structured and organised training pathway’.^1^ This was achieved by an adherence to an explicit curriculum for that specialty, regular assessments of progress and a Certificate of Completion of Training (CCT) date that defined the end point of specialist training. This reduced the time in training grades from an average of 12 years to a minimum of 7 years.^2^ Therefore, in theory, more trainees could achieve independent clinical competence earlier, allowing a greater proportion of consultants to provide care for forecasted vacancies. However, concerns were raised due to reduced operating volume due to adherence to CCT dates,^3^ variability in assessments, lack of experience before consultancy and therefore patient safety.

In 2005, Sir Liam Donaldson introduced further changes to training by implementing the Modernising Medical Careers (MMC) training curriculum.^4^ This markedly changed all postgraduate training in the UK to improve patient care. A two-year foundation programme was introduced, followed by broad-based basic specialist training programmes, which became the blueprint for core surgical training. At the same time, the European Working Time Directive (EWTD) reduced working hours to 56 hours per week in 2004, and then subsequently to 48 hours per week in 2009. The introduction of the MMC and EWTD translated into less ‘hands on’ experience for surgical trainees.

Pre-Calmanisation training hours equated to approximately 30,000 hours before CCT, but following the introduction of EWTD it fell to 6,000 hours.^5^ It is difficult to postulate that surgical training hours translates directly into useful operative experience. However, the amount of emergency^3,6^ and elective^7^ general surgery being carried out by trainees has reduced significantly over time, particularly after implementation of the EWTD.^3,8^

Current evidence indicates that colorectal surgeons operating on higher volumes of cancer patients have improved patient outcomes significantly.^9^ The concern following the shortening of colorectal surgery training in the UK and the reduced number of hours in training is that there will be fewer opportunities to hone operative prowess, potentially impacting patients negatively.

The aim of this study was to evaluate whether differences in a colorectal consultant's training impacted on their risk-adjusted 90-day postoperative mortality rate (%, AMR) for elective colorectal cancer (CRC) patients in England.

## Methodology

This retrospective cohort study analysed surgeon 90-day mortality data freely available in the public domain on the Association of Coloproctology of Great Britain and Ireland (ACPGBI) website. The data were taken from the Clinical Outcome Publication (COP) of 2016. COP data are taken from data submitted by 146 NHS Trusts in England to the National Bowel Cancer audit. Outcomes data are risk adjusted. As this was an observational study using publicly available and anonymous patient information, ethical approval was not required.

Adults (≥18 years of age) whose CRC was diagnosed between 2010 and 2015, in England, who subsequently went on to have an elective major resection under a named consultant were included in the COP 2016 and thus this study.

**Figure 1:**
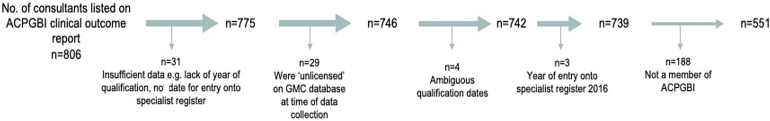
Flow diagram of exclusion criteria. ACPGBI = Association of Coloproctology of Great Britain and Ireland; GMC = General Medical Council

Initially, 806 colorectal consultants were registered on the COP 2016 database (Figure 1). However, consultants were excluded if they were ‘unlicensed’ or had insufficient data on their year of entry onto the General Medical Council (GMC) specialist register for general or colorectal surgery. Consultants were excluded from the study if they had been a consultant for less than one year to limit confounding the results through a lack of procedures or experience. Non-ACPGBI members were also excluded. Members of a professional body are more likely to be involved improving skills and involved in research, which could confound surgeon outcome mortality rates.^10^

A total of 51,562 patients registered under 551 consultants met the inclusion and exclusion criteria.

### Independent variable

In the UK, completion of formal postgraduate medical training is recognised by the CCT, awarded by Royal Colleges and the GMC. The CCT allows a clinician to enter the GMC specialist register and permits them to apply to consultant posts.

The independent variable in this study was the curriculum under which the consultant completed their CCT. Through the GMC specialist register, a consultant's CCT year was used to group them into 2 cohorts:

1. Pre-Calman-trained consultants (pre-CTrs): those whom completed their training before 1998.
2. Post-Calman-trained consultant (post-CTrs): this combined 2 groups:
  a) Consultants receiving their CCT under the Calman Training Principles, 1998–2007 (CTr)
  b) Consultants receiving their CCT under the Modernising Medical Careers Curriculum, 2008 and onwards (MMC).

### Dependent variable

The primary outcome measure was a risk-adjusted 90-day postoperative mortality rate.^11^ This was calculated by identifying all patients who died within 90 days after elective CRC surgery by a particular surgeon, out of their total elective cancer resection cases for patients diagnosed with CRC between 2010 and 2015.

The mortality rate was risk adjusted for case mix to accommodate for preoperative patient confounding variables that have the potential to penalise surgeons for undertaking resections on ‘high-risk’ cases. Factors included in the model are: age, gender, comorbidities guided by the Charlson Comorbidity Index,^12^ American Society of Anaesthesiologists grade, mode of admission, integrated clinicopathological stage (TNM 5^th^ edition)^13^ and site of CRC.

Data were also collected on the surgeon gender, GMC number, total number of major elective resection procedures and actual patient deaths during the data collection period. A trust (regional) 90-day AMR was also used, as patient outcomes can be affected by trust volume, resources and affiliation.

### Data analysis

Individual surgeon AMRs were non-Gaussian distributed data, calculated via skewness and kurtosis statistics models, and thus a Mann–Whitney *U* test was used to test for significance between cohorts. The Trust 90-day AMR was Gaussian distributed, therefore an independent sample *t*-test was used to test for significance for this independent variable. A chi-square test was used for categorical variables. *p* values <0.05 were considered statistically significant. Statistical analysis was performed using IBM SPSS Statistics version 22.0, Armonk, NY.

## Results

Table 1 illustrates differences between consultant cohorts in this study. Patients diagnosed with CRC between 2010 and 2015 undergoing an elective resection were operated on mainly by consultants trained post CTr (*n*=467). A total of 84 pre-CTrs were included in this study. Furthermore, the majority of consultants operating in this study were male, *n*=481 (87.3%). Pre-CTrs had a significantly lower proportion of female consultants, 3.6% (*n*=3) compared with post-CTr, 14.3% (*n*=67).

**Table 1:** Differences in consultant training and AMR

Consultant training	*n*	Female sex, % (*n*)	Median procedures, (IQR)	Mean AMR for trust, % (SEM)	Median AMR for surgeons, % (IQR)
Pre-CTr	84	3.6 (3)	104 (79, 138)	2.6 (0.08)	2.7 (1.7, 3.7)
Post-CTr	467	14.3 (67)	89 (61, 118)	2.7 (0.04)	2.1 (0.7, 3.6)

AMR = risk-adjusted 90 day post-operative mortality rate; IQR = interquartile range; pre-CTr = non-Calman Trained Consultants; post-CTr = Calman Trained and Modernising Medical Careers Trained Consultants; SEM = standard error of the mean

A Mann–Whitney *U* test revealed a statistically significant difference in the number of elective resections between the two consultant cohorts (*p*=0.008). Those completing their training more recently in the post-CTr cohort recorded a lower number of median colorectal resections (median=89 procedures, interquartile range, IQR 61–118 procedures) over the five-year collection period, when compared with pre-CTr cohort (median=104 procedures, IQR 79–138 procedures).

**Figure 2:**
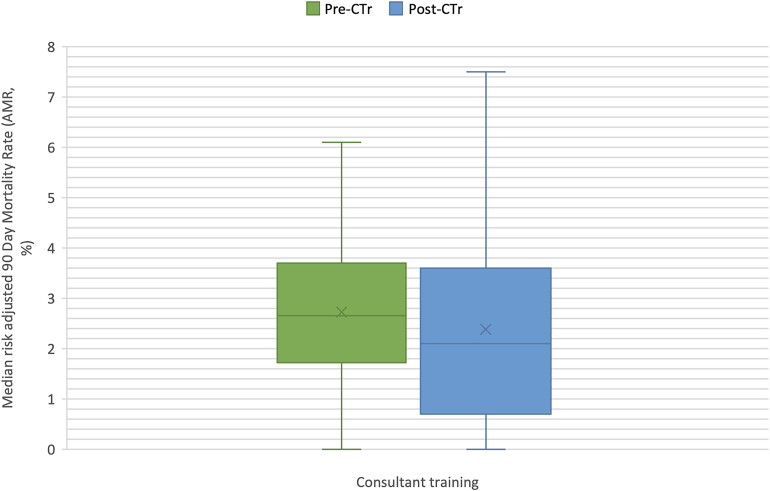
Boxplot diagram illustrating differences in consultant surgeon training and a risk-adjusted 90 day postoperative mortality rate. Outliers removed: Pre-CTr = 7.0, 8.0. Post-CTr = 8.5, 8.6, 10.1, 27.3. AMR = risk-adjusted 90-day postoperative mortality rate; pre-CTr = non-Calman Trained Consultants; post-CTr = Calman Trained and Modernising Medical Careers Trained Consultants.

The median AMR for all consultants in the study was 2.2% (IQR=2.8%). Analysis of the type of training received by consultants demonstrated that those trained under the post-CTr (median AMR=2.1%, IQR=2.9) had a significantly lower AMR when compared with pre-CTrs (median AMR=2.7%, IQR=2.0), *p*=0.022 (Figure 2). Meanwhile, there were no statistically significant differences between trust AMRs between different types of consultant training cohorts (*p*=0.414) (Table 1).

## Discussion

This study suggests that patients undergoing an elective CRC resection after being diagnosed between 2010 and 2015 had a significantly lower risk-adjusted 90-day mortality rate when operated on by consultants trained under the post-Calman curriculum (2.1%, IQR 2.9) compared with those completing training in the pre-Calman era (2.7%, IQR 2.0). Furthermore, the reduction in mortality is present despite a significantly lower number of median procedures carried out by post-CTr consultants in the same time period. This study has also demonstrated no significant difference between trust AMRs. Therefore, regional variation in treatment for CRC is unlikely to account for the differences in the AMR.

A reduction in trainee operative cases from the pre-Calman era to the MMC and post-EWTD era has already been established.^3,6–8^ As surgery is a craft specialty, theatre experience is essential. This has been highlighted in studies that support a positive volume outcome relationship for most operations,^14^ including in colorectal surgery.^9^ Moreover, an early retrospective study in America highlighted greater morbidity and mortality in complex alimentary surgery patients carried out by surgeons who had just completed residency training compared with more experienced surgeons.^15^ The findings from this study contradict this.

The improved patient outcomes by consultants trained in an era where less ‘hands on’ experience was gained than their more senior colleagues before CCT is perhaps a testament to the shorter, more structured and streamlined training. The Calman and subsequent MMC reforms moved away from apprenticeship to organised training programmes.^16^ Time constraints in the NHS, including the EWTD, have meant that these training programmes were instilled with supervision. Training authorities actively encouraged and supported consultant-led mentorship to tutor a nationally recognised curriculum,^17^ although it must be noted theatre time is often directed to meet trust goals as opposed to training despite the financial incentive.^3^ Structured mentorship programmes in laparoscopic colorectal surgery have shown similar outcomes between trainees and consultant trainers.^18^ This advocates that formal training can improve surgeon outcomes by negotiating the learning curves for procedures earlier, suggesting an explanation for the lower AMR for post-Calman trained consultants.

The contrast in the AMR among pre- and post-Calman consultants may have also been affected by the explosion of laparoscopic surgery during the study period.^19^ Post-Calman trainees were more likely to undergo dedicated laparoscopic fellowships in addition to training at a time when laparoscopic surgery was being recommended as an alternative to open resections for CRC.^20^ Fellowships have been associated with beneficial outcomes for patients, thus providing them with an unfair advantage.^21^ At the same time, the pre-Calman trainees were likely to have been learning laparoscopic CRC resections either independently or as part of the LapCo training programme.^20^ This could impact their AMR unfairly, as this cohort were traversing the learning curve for a new technique.

As mentioned previously, high-volume colorectal surgery is linked to improved patient outcomes.^9^ For patients with CRC diagnosed between 2010 and 2015, more senior CTrs and nCTrs carried out more operations for these patients compared with MMC consultants. On average, they undertook 15 more elective major resections. ACPGBI previously recommend a minimum of 20 resections with curative intent per annum per consultant.^22^ According to this study, MMC consultants may not be reaching this threshold. This requires further investigation. It could be speculated that newly accredited consultants may be more selective in cases, while senior consultants are taking on more difficult cases, hence the greater AMR and greater number of median cases. This is despite the mortality rate being risk adjusted for case mix.^11^ Cases were also performed in the era of publication of clinician-reported outcomes. Thus, the post-CTr surgeons may have been influenced to undertake risk-averse strategies, influencing their AMR.

The AMR model used in the COP and this study conveyed a wide range of patient and tumour-related factors with a large population sample. However, use of an AMR alone could be criticised in this study. Key factors not highlighted include preoperative chemoradiotherapy and type of surgical access. These could impact outcomes and should be included in the study. Furthermore, the AMR is not necessarily reflective of the consultant surgeon's skill alone; there is a multidisciplinary team providing treatment care. The care of the anaesthetist, critical care doctors, doctors in training, physiotherapists, nurses and many more play an invaluable role in patient outcomes. Finally, elective surgery mortality rates are low, and this can make it insensitive for identifying performance and many failings do not always cause death.^23^

Other outcomes such as surgical margins, stoma reversal rates, 30-day unplanned readmission rates and postoperative reoperation rates could be used in conjunction with the AMR to reflect a more sensitive marker of surgical performance. Other points to consider are that, regardless of consultant training, elective cancer resections make only a proportion of a surgeon's overall work; emergency and noncancer patients were excluded from the study. Therefore, to gain a wider perspective of surgeon performance, these patients should be included. It may be that the more complex procedures are carried out by consultants who have been in post longer.

Finally, the experience gained in the years after practising as an independent consultant surgeon may dilute some of the influence of training. This is important for surgeons who had an early CCT date, particularly those in the pre-CTr cohort.

## Conclusion

This study demonstrates that patients undergoing an elective CRC resection in 2010–2015 in England have a significantly lower AMR when operated on by post-CTr consultants compared with pre-CTr consultants despite a lower volume of operating experience. Further scope for investigation includes the acknowledgement of dedicated laparoscopic surgery fellowships or participation in the LapCo programme, and use of a wider range of dependent variables, over a longer time period including emergency and noncancer patients.
